# Long-Term Treatment with the Sodium Glucose Cotransporter 2 Inhibitor, Dapagliflozin, Ameliorates Glucose Homeostasis and Diabetic Nephropathy in *db/db* Mice

**DOI:** 10.1371/journal.pone.0100777

**Published:** 2014-06-24

**Authors:** Naoto Terami, Daisuke Ogawa, Hiromi Tachibana, Takashi Hatanaka, Jun Wada, Atsuko Nakatsuka, Jun Eguchi, Chikage Sato Horiguchi, Naoko Nishii, Hiroshi Yamada, Kohji Takei, Hirofumi Makino

**Affiliations:** 1 Department of Medicine and Clinical Science, Okayama University Graduate School of Medicine, Dentistry and Pharmaceutical Sciences, Okayama, Japan; 2 Department of Diabetic Nephropathy, Okayama University Graduate School of Medicine, Dentistry and Pharmaceutical Sciences, Okayama, Japan; 3 Department of Neurochemistry, Okayama University Graduate School of Medicine, Dentistry and Pharmaceutical Sciences, Okayama, Japan; Hirosaki University Graduate School of Medicine, Japan

## Abstract

Inhibition of sodium glucose cotransporter 2 (SGLT2) has been reported as a new therapeutic strategy for treating diabetes. However, the effect of SGLT2 inhibitors on the kidney is unknown. In addition, whether SGLT2 inhibitors have an anti-inflammatory or antioxidative stress effect is still unclear. In this study, to resolve these issues, we evaluated the effects of the SGLT2 inhibitor, dapagliflozin, using a mouse model of type 2 diabetes and cultured proximal tubular epithelial (mProx24) cells. Male *db/db* mice were administered 0.1 or 1.0 mg/kg of dapagliflozin for 12 weeks. Body weight, blood pressure, blood glucose, hemoglobin A1c, albuminuria and creatinine clearance were measured. Mesangial matrix accumulation and interstitial fibrosis in the kidney and pancreatic β-cell mass were evaluated by histological analysis. Furthermore, gene expression of inflammatory mediators, such as osteopontin, monocyte chemoattractant protein-1 and transforming growth factor-β, was evaluated by quantitative reverse transcriptase-PCR. In addition, oxidative stress was evaluated by dihydroethidium and NADPH oxidase 4 staining. Administration of 0.1 or 1.0 mg/kg of dapagliflozin ameliorated hyperglycemia, β-cell damage and albuminuria in *db/db* mice. Serum creatinine, creatinine clearance and blood pressure were not affected by administration of dapagliflozin, but glomerular mesangial expansion and interstitial fibrosis were suppressed in a dose-dependent manner. Dapagliflozin treatment markedly decreased macrophage infiltration and the gene expression of inflammation and oxidative stress in the kidney of *db/db* mice. Moreover, dapagliflozin suppressed the high-glucose-induced gene expression of inflammatory cytokines and oxidative stress in cultured mProx24 cells. These data suggest that dapagliflozin ameliorates diabetic nephropathy by improving hyperglycemia along with inhibiting inflammation and oxidative stress.

## Introduction

Diabetic nephropathy is a leading cause of chronic renal failure in western world [1]. In the past, several mechanisms have been suggested to involve in the initiation and deterioration of diabetic nephropathy, including hemodynamic and genetic factors, intracellular metabolic anomalies, and advanced glycation end products [2]. Emerging evidence suggests that inflammation is crucially contributed in the pathophysiology of diabetic nephropathy [3]. Recently, many studies have also suggested that production of reactive oxygen species (ROS) is enhanced by hyperglycemia, and oxidative stress has been involved in the onset and progression of diabetic nephropathy [4]. Therefore, the regulation of inflammation and oxidative stress could be a potential target in the treatment of diabetic nephropathy.

Sodium glucose cotransporter 2 (SGLT2), that is located on the apical side of the proximal tubular cells, can transport sodium and glucose concurrently within the proximal tubules [5]. Under normoglycemic conditions, SGLT2 can reabsorb about 90% of the glucose in the early segments of the proximal tubules [6]. In recent years, SGLT2 inhibitors, which can inhibit reabsorption of filtered glucose by blocking SGLT2, have been developed and proposed as novel hypoglycemic agents for treating patients with diabetes mellitus [7]. A large number of SGLT2 inhibitors have been developed, and numerous basic and clinical studies have been executed in the last decade [8]. Although SGLT2 inhibitors are novel and promising drugs for treating type 2 diabetes patients, the effect of SGLT2 inhibition on diabetic nephropathy is unknown.

Dapagliflozin is a very selective and potent SGLT2 inhibitor [9], and is the first-in-class SGLT2 inhibitor launched on the market in 2012 [10]. Various clinical studies have shown improvements in glycemic control with both monotherapy and combination therapy of dapagliflozin [11]. In addition, dapagliflozin was associated with additional non-glycemic benefits including reduction in blood pressure and body weight in most clinical trials [12]. Although several studies with animal models suggest that long-term administration of SGLT2 inhibitors, including dapagliflozin, preserves pancreatic β-cell function with improved glucose homeostasis [9], [13], [14], [15], the influences of SGLT2 inhibition on diabetic nephropathy and renal function have not been elucidated.

The purpose of this study was to investigate the hypothesis that inhibition of SGLT2 by dapagliflozin ameliorates glucose homeostasis while preserving β-cell mass, and retards the progression of diabetic nephropathy by inhibiting inflammation and oxidative stress in a mouse model of type 2 diabetes and obesity.

## Materials and Methods

### Animal Care and Experiments

We purchased six-week-old male diabetic *db/db* mice (BKS.Cg-*lepr^db^/lepr^db^*) and non-diabetic *db/m* mice (BKS.Cg-*lepr^db^/*+) from CLEA Japan (Tokyo, Japan). All mice were kept in light-controlled room and allowed free access to tap water and food. Dapagliflozin was kindly supplied by Bristol-Myers Squibb (Pennington, NJ, USA). Dapagliflozin (0.1 or 1.0 mg/kg/day) was administrated to *db/db* mice (*n* = 6) by gavage for 12 weeks starting at the age of 8 weeks. Control *db/db* mice (*n* = 5) and control *db/m* mice (*n* = 5) received saline for 12 weeks. The mice were anesthetized by an injection of pentobarbital at 20 weeks of age. After the mice were sacrificed by exsanguination through cutting cervical artery under anesthesia, the kidneys were removed and weighed. The kidneys and pancreas were processed as previously described [16]. The study protocol was approved by the Animal Ethics Review Committee of Okayama University (OKU-2012356). All animal care and procedures were performed in accordance with the Guidelines for Animal Experimentation at Okayama University, the Japanese Government Animal Protection and Management Law, and the Japanese Government Notification on Feeding and Safekeeping of Animals.

### Metabolic Data

Body weight was measured weekly. Blood pressure, plasma glucose, urinary glucose and 24-h urinary albumin excretion (UAE) were measured every 4 weeks. Blood pressure was measured by the tail-cuff method (Softron, Tokyo, Japan). Plasma glucose and blood pressure were measured after an overnight fast. Hemoglobin A1c (HbA1c), water intake, food intake, kidney weight, blood urea nitrogen (BUN), creatinine and creatinine clearance (Ccr) were measured at the age of 20 weeks. Serum and urinary creatinine were measured using an enzymatic method (PUREAUTOS CRE-L, Shimizu Medical, Tokyo, Japan). HbA1c and UAE were measured as described previously [17].

### Histology

Tissue sections were cut from the paraffin-embedded kidney samples taken at 20 weeks of age and subjected to PAM and Masson trichrome staining. All tissue sections were examined using a BZ-9000 microscope (Keyence, Osaka, Japan). The mesangial matrix index (MMI) was evaluated using BIOZERO software (Keyence) as previously described [16]. To determine the MMI, 10 randomly selected glomeruli in the cortex per animal were evaluated under high magnification (×400).

### Immunofluorescent Staining

Immunofluorescent staining of kidney and pancreas was performed as previously described [18]. Briefly, renal expression of type IV collagen was detected a rabbit antibody for type IV collagen (Millipore, Temecula, CA, USA) followed by Alexa Fluor 488 goat anti-rabbit IgG (Invitrogen, Carlsbad, CA, USA). Similarly, pancreatic β-cells were detected using guinea pig anti-insulin (Abcam, Cambridge, UK) followed by Alexa Fluor 488 goat anti-guinea pig IgG (Invitrogen). The positive area of type IV collagen in the glomerulus was calculated in the same way as MMI. The proportion of β-cells in the pancreatic tissue was calculated using BIOZERO software (Keyence). The insulin-positive area relative to the area of the whole pancreatic tissue was analyzed in more than 100 islets per group.

### Immunoperoxidase Staining

Immunoperoxidase staining was performed as previously described [17]. In brief, macrophage infiltration was analyzed using a monoclonal antibody for murine monocyte/macrophage (F4/80, Abcam), followed by HRP-conjugated goat anti-rat IgG antibody (Millipore). The number of F4/80-positive cells was calculated in 10 glomeruli and intestitia per animal, and the mean number of F4/80 positive cells per glomerulus and interstitial tissue (number per mm^2^) was used for the estimation.

NADPH oxidase 4 (Nox4) immunoperoxidase staining was performed as described previously [19]. Briefly, renal tissues were stained with Nox4 rabbit antibody (Novus Biologicals, Littleton, CO, USA) for 12 h at 4°C followed by HRP-conjugated goat anti-rabbit IgG antibody (Millipore). The proportion of the area stained with Nox4 antibody of the total area was calculated using BIOZERO software (Keyence). To quantify the proportional area of staining, 10 views of the renal cortex were randomly selected in each slide.

### Quantitative Analysis of Gene Expression in the Renal Cortex

RNA was isolated from the renal cortex of 20-week-old mice as described previously [16]. To determine the expression of *CD14*, *CD11c*, *CD206*, *transforming growth factor* (*TGF*)-*β*, *intercellular adhesion molecule* (*ICAM*)-*1*, *monocyte chemoattractant protein* (*MCP*)-*1*, *osteopontin*, *caspase-12* and *Bax* in the renal cortex, quantitative RT-PCR (qRT-PCR) was performed as described previously [16]. Each sample was normalized against *Atp5f1* mRNA expression and analyzed in triplicate.

### ROS Expression

To evaluate the effect of dapagliflozin on ROS production, superoxide anion radicals were detected by dihydroethidium (DHE) staining (Molecular Probes, Eugene, OR, USA). Briefly, the kidney sections were incubated with DHE (2 µmol/l) at 37°C in a humidified chamber protected from light for 45 min. The DHE fluorescence intensity was analyzed using BIOZERO software (Keyence) in 10 intestitia per animal.

### Terminal Transferase-mediated dUTP Nick-End Labeling (TUNEL) Assay

To evaluate the effect of dapagliflozin on apoptosis, kidney samples were incubated with an *in situ* apoptosis detection kit (Takara Bio) according to the manufacturer’s protocol. The mean number of TUNEL-positive cells in interstitia (number per mm^2^) was determined by observing more than 10 interstitia from each section.

### *In vitro* Experiments

Murine proximal tubular epithelial (mProx24) cells, kindly provided by Dr. Takeshi Sugaya (CMIC Co., Tokyo, Japan), were used as previously described [18]. DHE staining and qRT-PCR were performed as described above.

### Statistical Analysis

All data were expressed as mean ± SEM. Statistical analysis between groups was performed using one-way ANOVA followed by Scheffe’s test. A *P* value<0.05 was considered statistically significant.

## Results

### Effect of Dapagliflozin on Body Weight, Hyperglycemia and Renal Function

**Figure 1:**
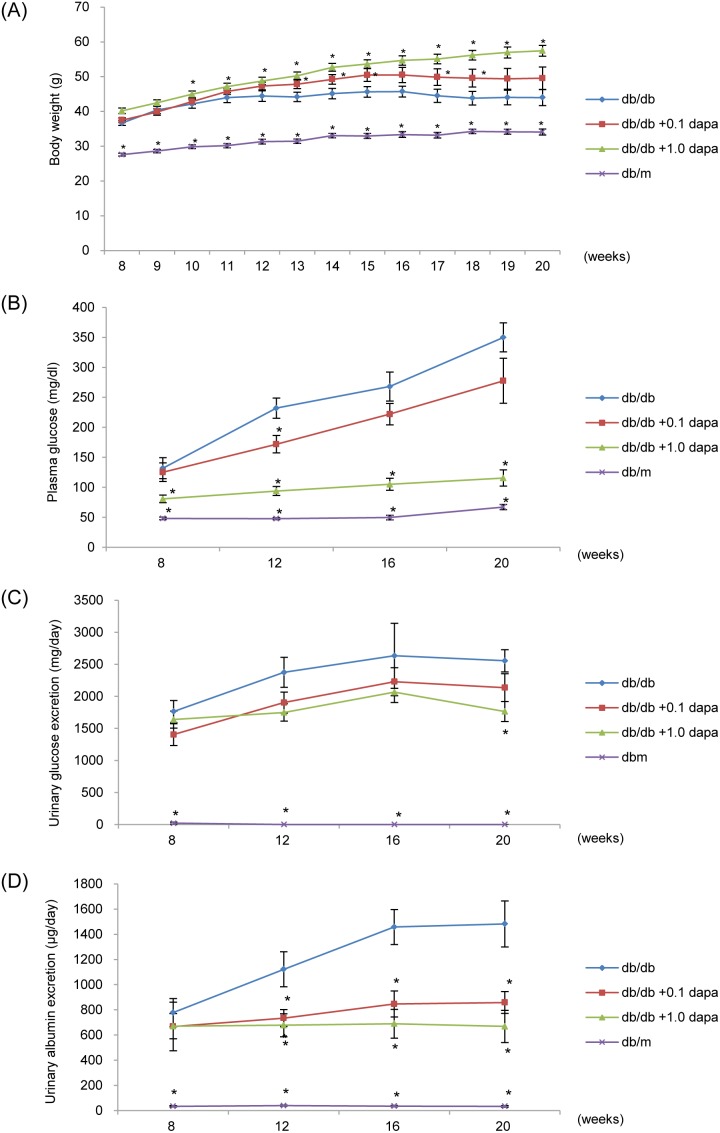
Effect of dapagliflozin on body weight, hyperglycemia and urinary albumin excretion (UAE). (A) Body weight was higher in the *db/db* group than in the *db/m* group during the study. Body weight in the *db/db* with 1.0 mg/kg dapagliflozin group (*db/db*+0.1 dapa group) was higher than in the *db/db* group from 10 to 20 weeks of age. Data are mean ± SEM. *P<0.05. (B–D) Plasma and urinary glucose, and UAE progressively increased in the *db/db* group during the 12-week observation period. These parameters were significantly lower in the *db/db*+1.0 dapa group than in the *db/db* group. Data are mean ± SEM. *P<0.05.

Body weight was higher in the *db/db* groups than in the *db/m* group during the study, and body weight in the *db/db* group treated with 0.1 or 1.0 mg/kg/day of dapagliflozin (*db/db*+0.1 dapa group and *db/db*+1.0 dapa group, respectively) was higher than in the *db/db* group from 10 to 20 weeks of age (Fig. 1A). Plasma and urinary glucose excretion progressively increased in the *db/db* groups during the study. However, dapagliflozin significantly reduced plasma and urinary glucose, and HbA1c compared with those in the *db/db* group at 20 weeks of age (Fig. 1B, 1C and Table 1). There were no significant differences in systolic and diastolic blood pressure between the four groups at 20 weeks of age. In addition, there were no significant differences in water and food intake between the *db/db*, the *db/db*+0.1 dapa and the *db/db*+1.0 dapa groups (Table 1).

**Table 1:** Influence of dapagliflozin on physiologic and metabolic parameters in *db/db* and *db/m* mice at 20 weeks.

	*db/m*	*db/db*	*db/db*+0.1 dapa	*db/db*+1.0 dapa
Systolic blood pressure (mmHg)	120.0±5.2	116.6±4.5	121.2±2.3	115.2±4.5
Diastolic blood pressure (mmHg)	79.4±3.2	78.8±2.3	86.3±1.6	84.3±3.0
HbA1c (%)	4.0±0.1	9.2±0.2^a^	8.5±0.3^a^	6.6±0.2^abc^
Water intake (ml/day)	4.8±0.4	31.1±4.1^a^	22.3±2.9^a^	19.8±1.9^a^
Food intake (g/day)	3.2±0.1	4.5±0.7	4.8±0.3	6.1±0.3^a^

*db/m*, nondiabetic control mice; *db/db*, untreated diabetic mice; *db/db*+0.1 dapa, dapagliflozin (0.1 mg/kg)-treated diabetic mice; *db/db*+1.0 dapa, dapagliflozin (1.0 mg/kg)-treated diabetic mice; HbA1c, hemoglobin A1c. Data are presented as mean ± SEM;

a *P*<0.05 vs. *db/m*,

b *P*<0.05 vs. *db/db*,

c *P*<0.05 vs. *db/db*+0.1 dapa.

UAE, a characteristic feature of diabetic nephropathy, progressively increased in the *db/db* group during this study. However, dapagliflozin decreased the UAE compared with that in the *db/db* group from 12 to 20 weeks of age significantly (Fig. 1D). The other parameters are summarized in Table 2. There were no significant differences in BUN and serum creatinine between the four groups at 20 weeks of age. Kidney weight and relative kidney weight were lower in the *db/db* groups than in the *db/m* group significantly, but there were no significant differences between the *db/db*, the *db/db*+0.1 dapa and the *db/db*+1.0 dapa group. We speculate that the kidney became atrophic in the *db/db* mice, because of the continuous high-glucose level. Ccr was higher in the *db/db* group and the *db/db*+0.1 dapa group than in the *db/m* group, but there were no significant differences between the *db/db*, the *db/db*+0.1 dapa, and the *db/db*+1.0 dapa groups.

**Table 2:** Influence of dapagliflozin on renal structural and functional parameters at 20 weeks.

	*db/m*	*db/db*	*db/db*+0.1 dapa	*db/db*+1.0 dapa
Kidney weight (mg)	379.0±39.6	247.0±6.8^a^	239±9.4^a^	252.5±9.1^a^
Relative kidney weight (mg/g body weight)	11.5±1.0	6.0±0.3^a^	5.2±0.4^a^	4.5±0.1^a^
BUN (mg/dl)	20.3±0.7	29.1±2.8	24.6±2.5	25.9±0.6
Serum creatinine (mg/dl)	0.10±0.01	0.12±0.02	010±0.01	0.12±0.02
Urine volume (ml/day)	1.0±0.1	23.4±3.1^a^	19.2±2.4^a^	16.3±1.9^a^
Ccr (ml/min)	4.80±0.54	9.42±0.96^a^	9.81±0.78^a^	6.40±0.65^c^

*db/m*, nondiabetic control mice; *db/db*, untreated diabetic mice; *db/db*+0.1 dapa, dapagliflozin (0.1 mg/kg)-treated diabetic mice; *db/db*+1.0 dapa, dapagliflozin (1.0 mg/kg)-treated diabetic mice; BUN, blood urea nitrogen; Ccr, creatinine clearance. Data are presented as mean ± SEM;

a *P*<0.05 vs. *db/m*,

b *P*<0.05 vs. *db/db*,

c *P*<0.05 vs. *db/db*+0.1 dapa.

### Dapagliflozin Suppresses Mesangial Matrix Accumulation and Interstitial Fibrosis

**Figure 2:**
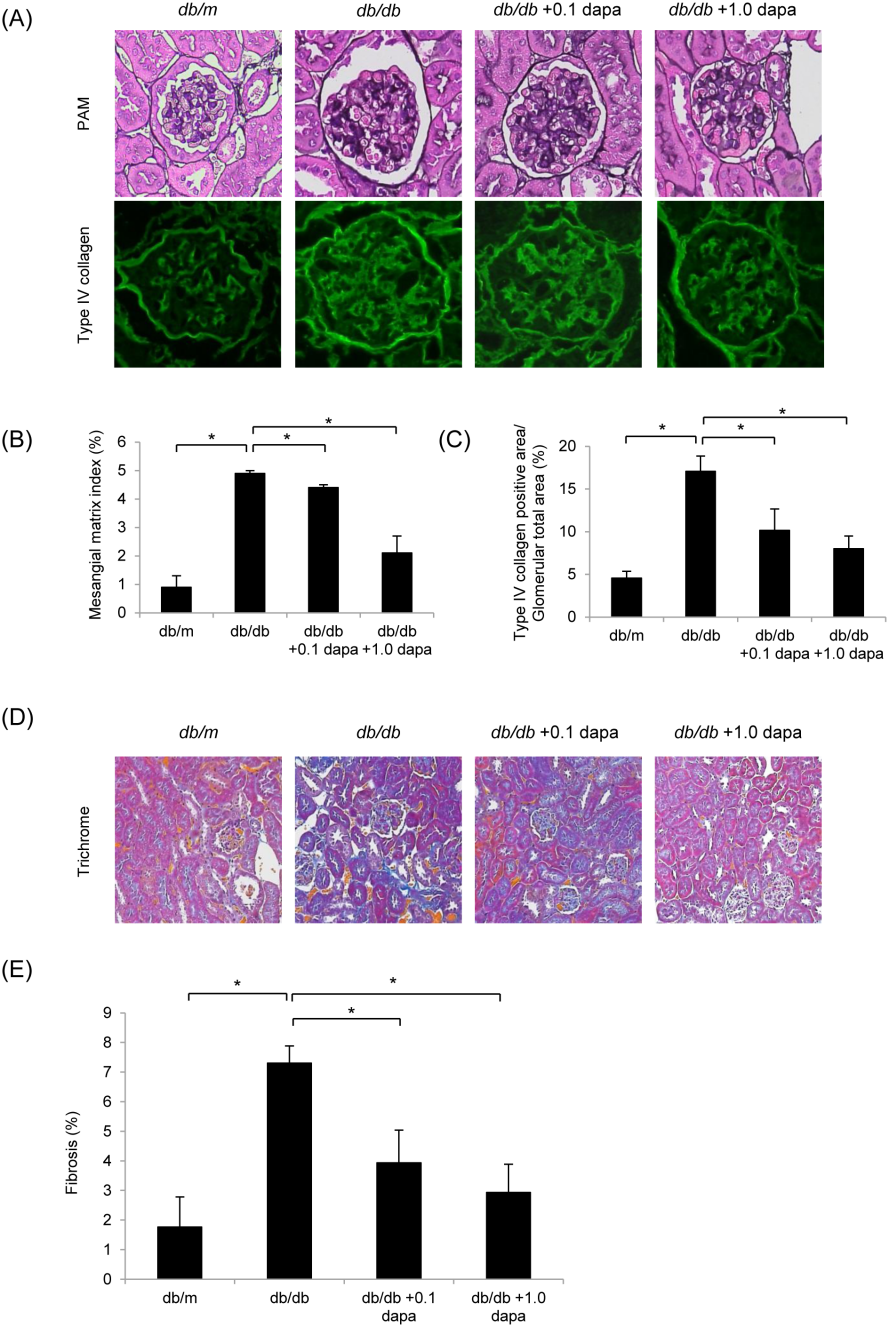
Dapagliflozin suppresses mesangial matrix accumulation and interstitial fibrosis. (A) Periodic acid-methenamine silver (PAM) and type IV collagen staining of kidney sections. Mesangial matrix accumulation was prominent in the *db/db* group. Dapagliflozin suppressed the increase in mesangial matrix accumulation compared with that in the *db/db* group. Original magnification, ×400. (B) Mesangial matrix index of the glomeruli. Data are mean ± SEM. *P<0.05. (C) Type IV collagen positive area in the glomeruli. Data are mean ± SEM. *P<0.05. (D) Masson’s trichrome staining of kidney sections. Interstitial fibrosis was significantly higher in the *db/db* group than in the *db/m* group, and significantly lower in the *db/db*+1.0 dapa group than in the *db/db* group. Original magnification, ×100. (E) Percentages of fibrosis in interstitia. Data are mean ± SEM. *P<0.05.

As shown by PAM and type IV collagen staining (Fig. 2A), mesangial matrix accumulation was detected in the *db/db* group at 20 weeks of age. However, this outcome was ameliorated in the *db/db*+1.0 dapa group compared with that in the *db/db* group, as demonstrated by a reduction in the MMI from 4.9±0.1% in the *db/db* group to 2.1±0.6% in the *db/db*+1.0 dapa group (*P*<0.05; Fig. 2B). Immunofluorescent staining for type IV collagen showed the same tendency (Fig. 2C). Similarly, representative interstitia in the Masson’s trichrome-stained sections are shown in Fig. 2D. Interstitial fibrosis was significantly higher in the *db/db* group compared with that in the *db/m* group, and was suppressed in the *db/db*+0.1 dapa group and the *db/db*+1.0 dapa group (Fig. 2E). Collectively, these results demonstrate that administration of dapagliflozin ameliorates mesangial matrix expansion and interstitial fibrosis in *db/db* mice.

### Proinflammatory Macrophage Infiltration in the Kidney

**Figure 3:**
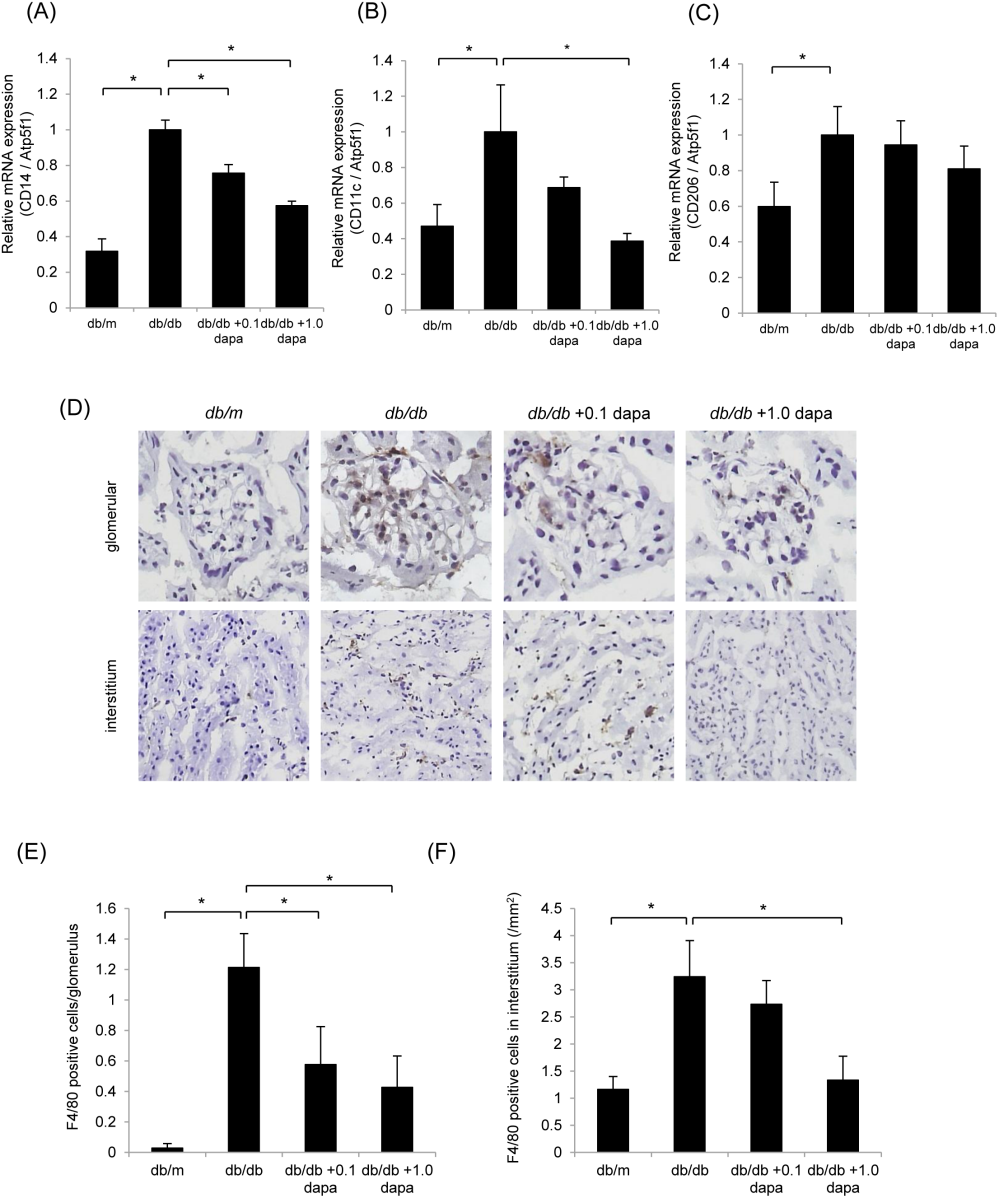
Dapagliflozin inhibits proinflammatory macrophage infiltration in the renal cortex. Quantitative RT-PCR analysis of the expression of *CD14* (A), *CD11c* (B) and *CD206* (C) showed that dapagliflozin suppressed gene expression in proinflammatory macrophages in the kidney. mRNA levels were normalized against *Atp5f1* expression. Data are mean ± SEM. *P<0.05. (D) Macrophage infiltration into the glomeruli and the interstitium was clearly evident in the *db/db* group compared with that in the *db/m* group, and was suppressed in the *db/db*+dapa groups compared with that in the *db/db* group. Original magnifications: ×400 for glomeruli and ×100 for interstitia. (E) Number of macrophages in the glomerulus. Data are mean ± SEM. *P<0.05. (F) Number of macrophages in the interstitia. Data are mean ± SEM. *P<0.05.

We performed qRT-PCR analysis to evaluate the macrophage infiltration into the kidney. Gene expression of *CD14*, a macrophage marker, was lower in the *db/db*+1.0 dapa group than in the *db/db* group (Fig. 3A). To distinguish which proinflammatory or anti-inflammatory macrophages are dominant in the kidney, we used primers for *CD11c* and *CD206*. CD11c is a marker for the proinflammatory (M1) subtype of macrophages, while CD206 is specific for the anti-inflammatory (M2) subtype of macrophages. The renal expression of *CD11c* was lower in the *db/db*+1.0 dapa group than in the *db/db* group (Fig. 3B); however, there were no differences in *CD206* between the *db/db*, the *db/db*+0.1 dapa and the *db/db*+1.0 dapa groups (Fig. 3C). To confirm these findings, we performed immunoperoxidase staining of F4/80, a marker for M1 macrophages. In both the glomeruli and interstitia, the number of macrophages were prominently increased in the *db/db* group compared with those in the *db/m* group (Fig. 3D). The macrophage infiltration into the glomeruli was significantly suppressed in the *db/db*+0.1 dapa and the *db/db*+1.0 dapa groups compared with the *db/db* group (Fig. 3D and 3E). Similarly, the macrophage infiltration into the interstitia was increased in the *db/db* group, but decreased in the *db/db*+1.0 dapa group (Fig. 3D and 3F). These results indicate that dapagliflozin suppresses proinflammatory macrophage infiltration in the kidney of *db/db* mice.

### Inflammatory Gene Expression in the Renal Cortex

**Figure 4:**
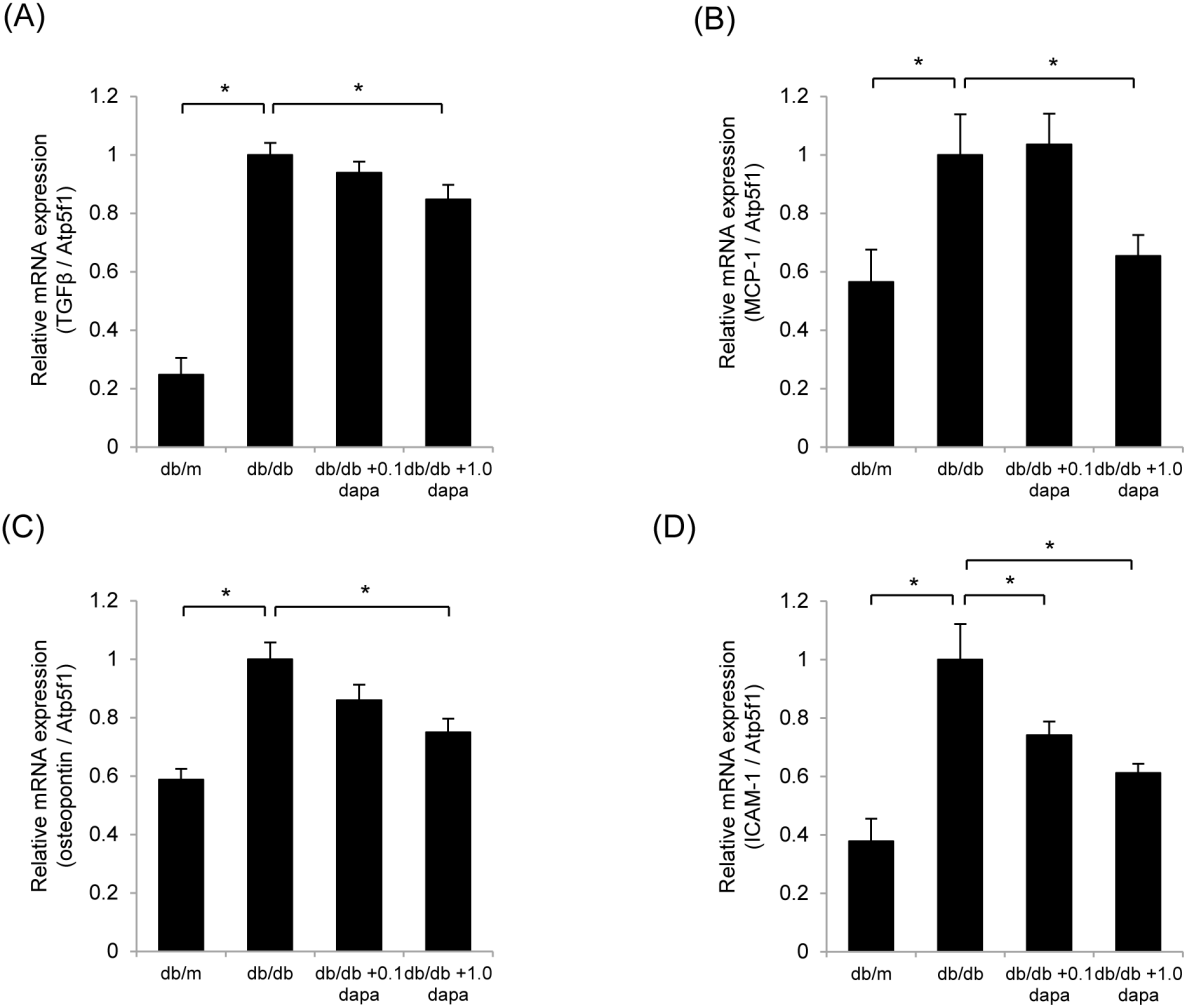
Dapagliflozin suppresses inflammatory gene expression in the renal cortex. Quantitative RT-PCR analysis of the expression of *TGF-β* (A), *MCP-1* (B), *osteopontin* (C) and *ICAM-1* (D) showed that dapagliflozin inhibited diabetes-induced inflammation in the kidney. mRNA levels were normalized against *Atp5f1* expression. Data are mean ± SEM. *P<0.05.

qRT-PCR analysis of kidney tissue revealed that the expression of several proinflammatory genes, including *TGF-β*, *MCP-1*, *osteopontin* and *ICAM-1*, was significantly increased in the *db/db* group, but suppressed by dapagliflozin in the *db/db*+1.0 dapa group (Fig. 4A–D).

### Oxidative Stress and Apoptosis in the Kidney

**Figure 5:**
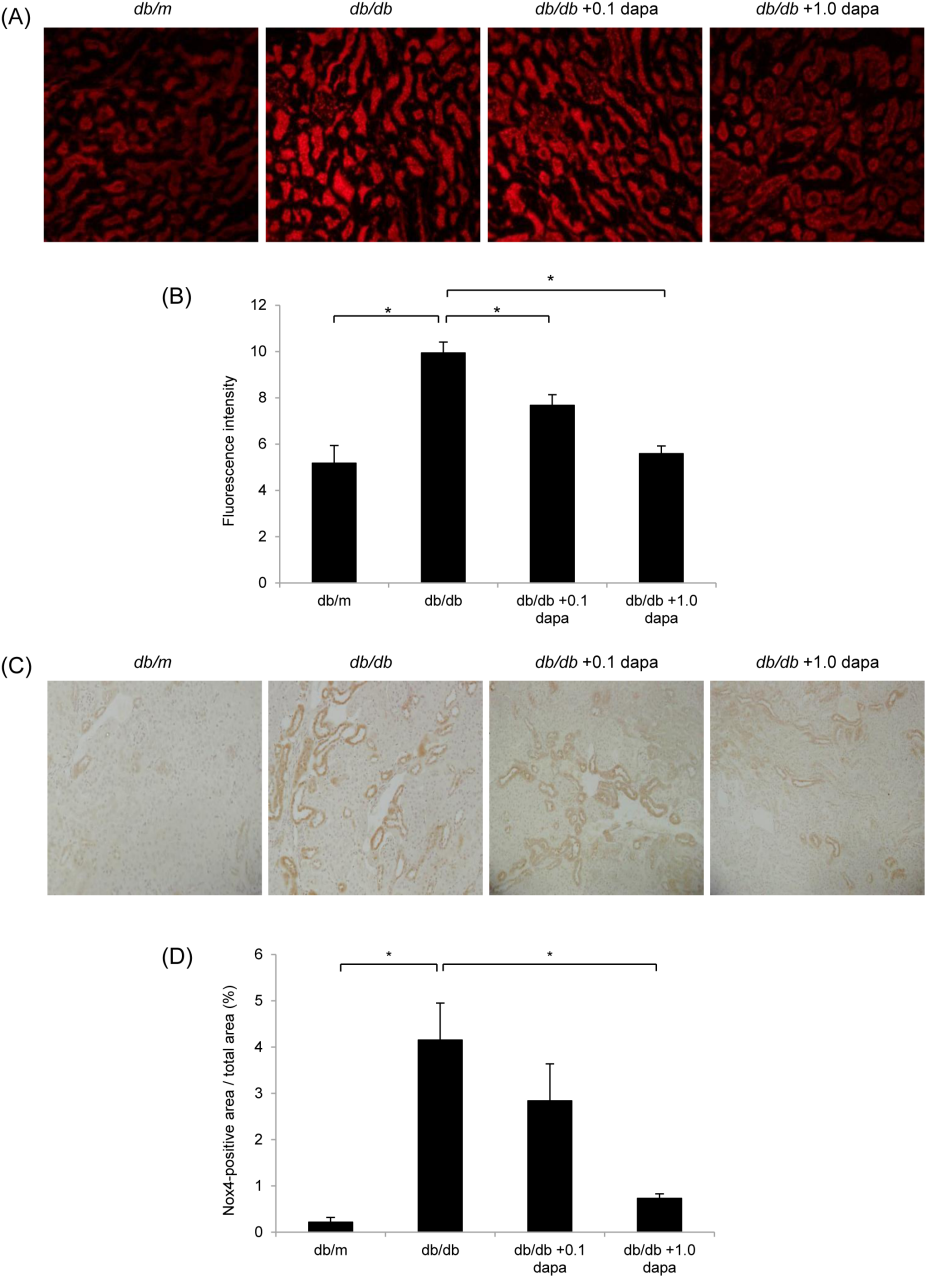
Dapagliflozin inhibits oxidative stress in the kidney. (A, B) ROS production was detected by fluorescence microscopy using dihydroethidium (DHE) staining. ROS was predominantly localized in the interstitia of *db/db* mice, and was suppressed in the *db/db*+dapa groups compared with that in the *db/db* group. Original magnification, ×100. Data are mean ± SEM. *P<0.05. (C, D) Localization of Nox4 was detected by immunohistochemistry. The expression of Nox4 was predominantly localized in the interstitia of *db/db* mice, and was suppressed in the *db/db*+dapa groups compared with that in in the *db/db* group. Original magnification, ×100. Data are mean ± SEM. *P<0.05.

**Figure 6:**
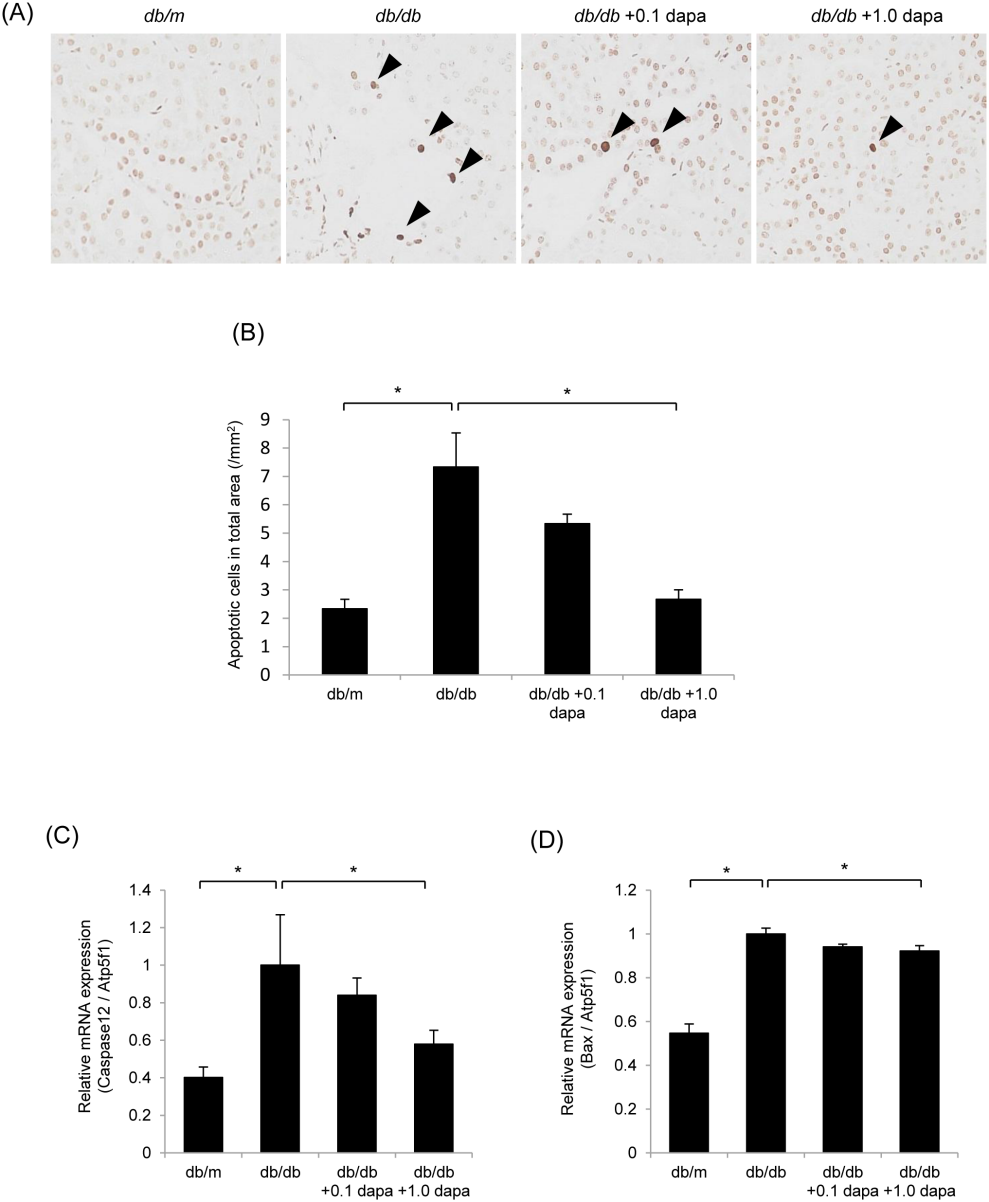
Dapagliflozin inhibits apoptosis in the kidney. (A, B) Apoptosis was detected by TUNEL staining. Arrowheads indicate the apoptotic nuclei. The number of apoptotic cells was higher in the interstitia of *db/db* mice than in *db/m* mice, and was lower in the *db/db*+dapa groups than in the *db/db* group. Original magnification, ×400. Data are mean ± SEM. *P<0.05. (C, D) Dapagliflozin reduced the mRNA levels of *Caspase-12* and *Bax* in the kidney. mRNA levels were normalized against *Atp5f1* expression. Data are mean ± SEM. *P<0.05.

To investigate the role of oxidative stress and apoptosis, and the effects of dapagliflozin on the pathogenesis of diabetic nephropathy, we conducted DHE staining, Nox4 immunostaining and the TUNEL assay on the kidney. ROS production, which was detected by DHE, was higher in the cortex of the *db/db* group than in that of the *db/m* group, but it was lower in the *db/db*+0.1 and *db/db*+1.0 dapa groups (Fig. 5A and B). Similarly, Nox4, a subunit of NADPH oxidase, was upregulated in the cortex of the *db/db* group, but its expression was attenuated in the *db/db*+1.0 dapa group (Fig. 5C and D). TUNEL staining confirmed that apoptosis was promoted in the *db/db* group, and that dapagliflozin markedly decreased the number of apoptotic cells (Fig. 6A and B). Furthermore, dapagliflozin markedly reduced the high gene expression of the proapoptotic factors, *Caspase-12* and *Bax*, in the *db/db* group (Fig. 6C and D). These data indicate that diabetes increases oxidative stress and apoptosis, and that oxidative stress and apoptosis are suppressed by dapagliflozin.

### Oxidative Stress and Inflammatory Gene Expression in Cultured Proximal Tubular Epithelial Cells

**Figure 7:**
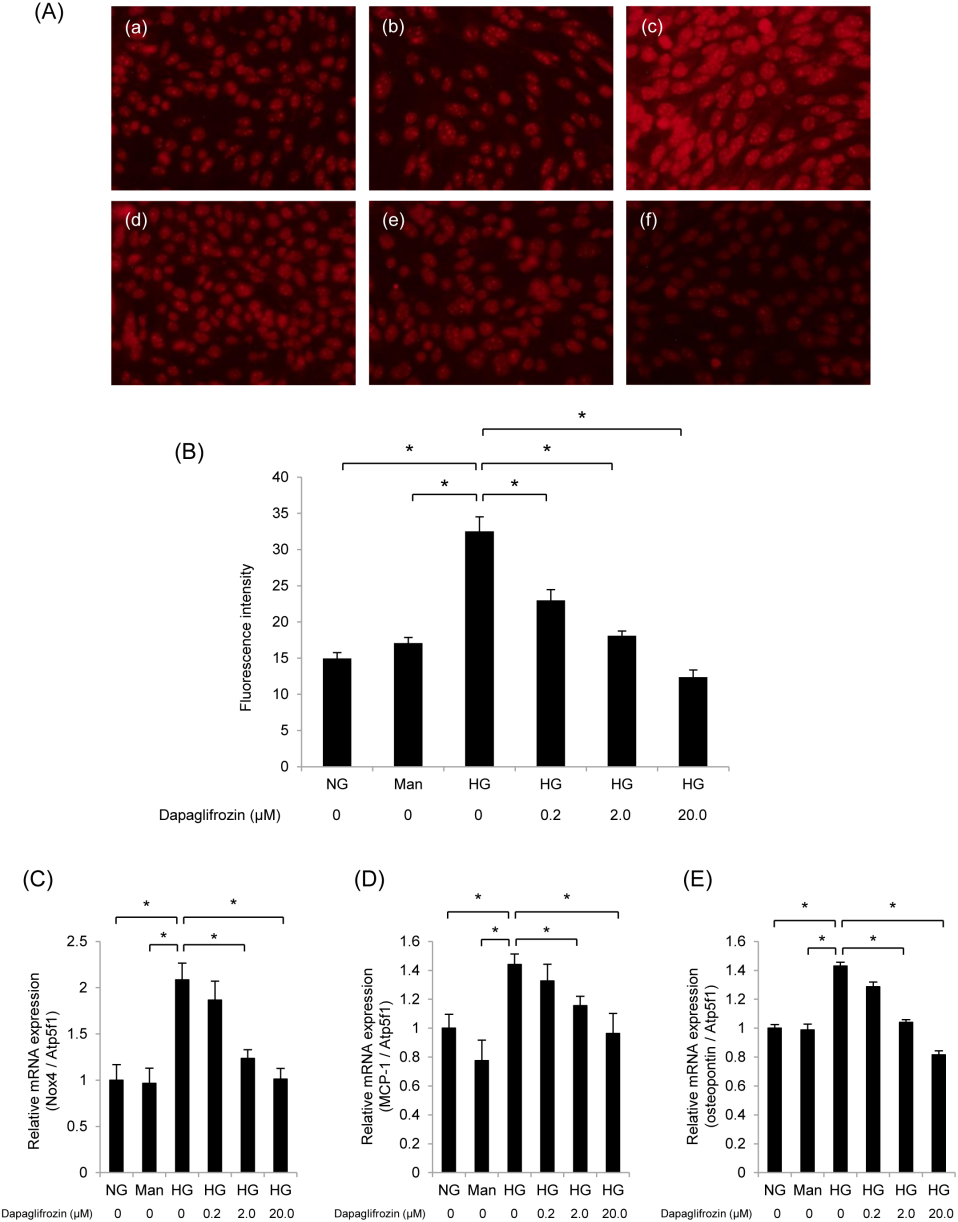
Dapagliflozin suppresses oxidative stress and inflammatory gene expression in cultured proximal tubular epithelial cells. (A) ROS production was detected by fluorescence microscopy using dihydroethidium (DHE) staining. ROS production was not increased by mannitol (b) compared with normal glucose (a), but was increased by high glucose (c). High-glucose-induced ROS production was decreased by dapagliflozin pretreatment in a dose-dependent manner (d: 0.2 nM; e: 2.0 nM; f: 20.0 nM). (B) Densitometric quantification of ROS production. Data are mean ± SEM. *P<0.05 vs. high glucose; NG: normal glucose; Man: mannitol; HG: high glucose; dapa: dapagliflozin. Quantitative RT-PCR analysis of the expression of *Nox4* (C), *MCP-1* (D) and *osteopontin* (E) showed that dapagliflozin inhibited diabetes-induced inflammation in the kidney. mRNA levels were normalized against *Atp5f1* expression. Data are mean ± SEM. *P<0.05.

To evaluate high-glucose-induced ROS production in cultured proximal tubular epithelial cells, we performed DHE staining. High-glucose medium increased ROS production in mProx24 cells, and dapagliflozin treatment significantly attenuated this increase (Fig. 7A and B). qRT-PCR analysis of mProx24 cells demonstrated that *Nox4* expression induced by high glucose stimulation was also suppressed by dapagliflozin (Fig. 7C). Similarly, the gene expression of *OPN* and *MCP-1* was upregulated by exposure to high glucose and attenuated by dapagliflozin (Fig. 7D and E). These findings suggest that dapagliflozin ameliorates oxidative stress and inflammation induced by high glucose in renal proximal tubular epithelial cells.

### Effect of Dapagliflozin on β-cell Mass in *db/db* Mice

**Figure 8:**
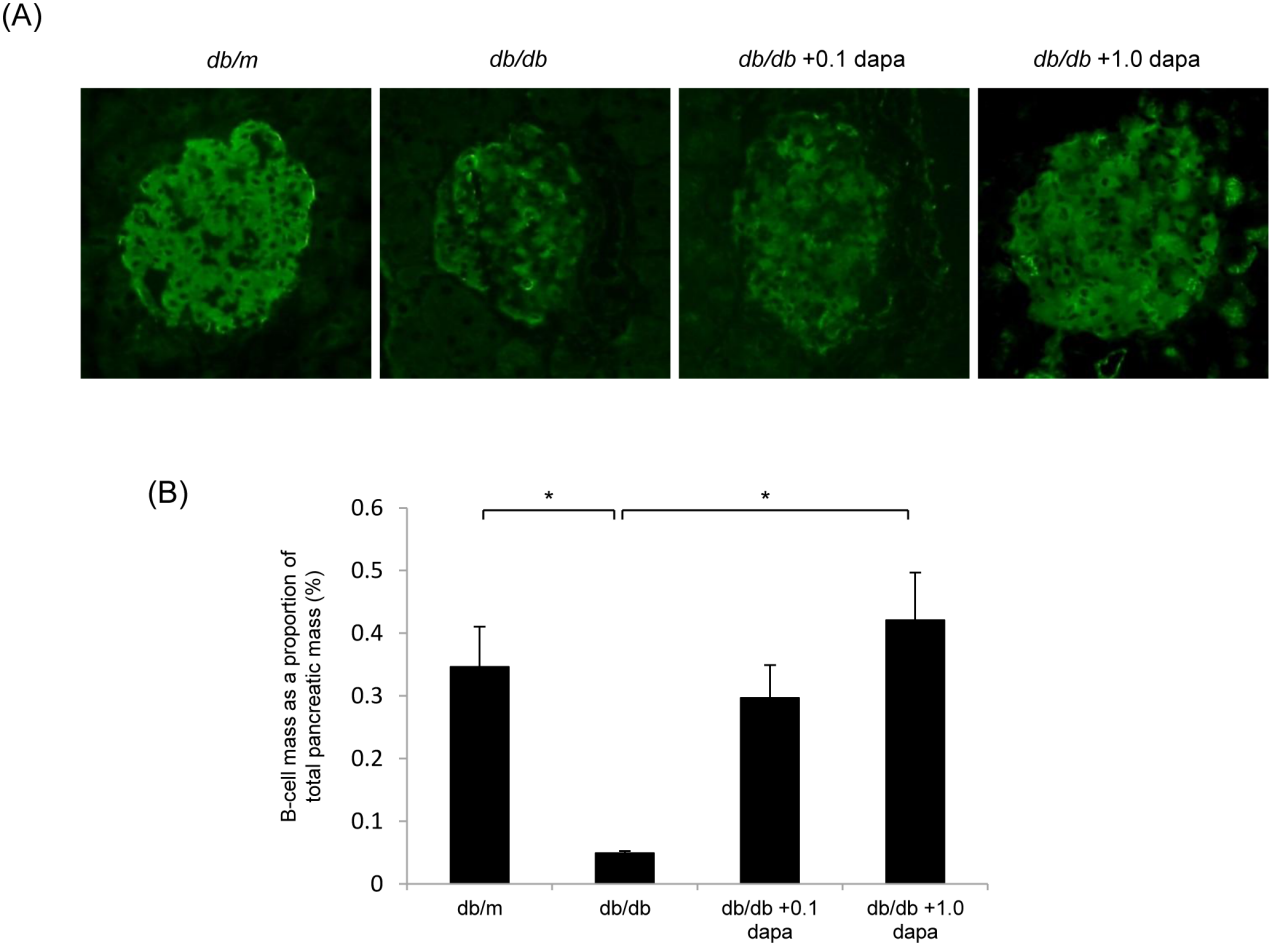
Treatment with dapagliflozin increases β-cell mass in *db/db* mice. (A) Representative immunofluorescent staining of insulin in pancreatic sections derived from *db/m*, *db/db*, and *db/db* with 0.1 or 1.0 mg/kg dapagliflozin mice. Original magnification, ×400. (B) The β-cell area is shown as a proportion of the area of the entire pancreas. Data are mean ± SEM. *P<0.05.

We evaluated the effect of dapagliflozin on β-cell morphology by immunoperoxidase staining of insulin (Fig. 8A). The β-cell mass was significantly lower in the *db/db* group compared with that in the *db/m* group at 20 weeks of age. However, dapagliflozin treatment significantly prevented the decrease in β-cell mass in a dose-dependent manner (Fig. 8B).

## Discussion

In the present study, we demonstrated that dapagliflozin, a novel SGLT2 inhibitor, suppressed hyperglycemia and restored β-cell mass in diabetic *db/db* mice. Administration of dapagliflozin reduced macrophage infiltration and the gene expression of inflammation, including *OPN*, *MCP-1* and *TGF-β* in the kidney of diabetic *db/db* mice. Moreover, oxidative stress and apoptosis were lower in the dapagliflozin-treated *db/db* mice than in the untreated mice. Our findings revealed that dapagliflozin exhibits potent antihyperglycemic effects and slows the progression of diabetic nephropathy.

Inhibitors of SGLT2 are newly developed antidiabetic agents and interfere the pathway of physiological glucose reabsorption in the kidney. At present, many preclinical and clinical studies of dapagliflozin, a selective SGLT2 inhibitor, have revealed that selective inhibition of SGLT2 is beneficial for type 2 diabetic patients independently of pancreatic β-cell function or insulin sensitivity, and that the kidney is a safe and effective target for treatment [20]. Although many studies in animals and humans have demonstrated that SGLT2 inhibitors reduce hyperglycemia measurements, including HbA1c, fasting and postprandial glucose, the effects of SGLT2 inhibitors on the organs are not well known. Several studies have demonstrated that genetic and pharmacological inhibition of SGLT2 preserve pancreatic β-cell function [15], [21], [22]; however, the effects of SGLT2 inhibitors on renal structures and function are not understood. Therefore, we investigated how dapagliflozin influences the progression of diabetic nephropathy using a mouse model of type 2 diabetes.

Inflammation is associated with the development of diabetic nephropathy, and targeting inflammation could be a therapeutic approach for the management of diabetic nephropathy [3], [23]. We have demonstrated that activation of nuclear hormone receptors, including peroxisome proliferator-activated receptor (PPAR) γ, PPARδ and liver x receptor, inhibits macrophage infiltration and inflammation, and ameliorates diabetic nephropathy in animal models [16], [24], [25]. In the present study, dapagliflozin decreased F4/80-positive macrophage infiltration into the kidney in a dose-dependent manner, and suppressed the gene expression of the proinflammatory M1 macrophage marker, *CD11c*, but not the anti-inflammatory M2 macrophage marker, *CD206*. Similarly, dapagliflozin suppressed the gene expression of the chemokine *MCP-1*, the adhesion molecule *ICAM-1*, and the cytokines *OPN* and *TGF-β*. Moreover, our *in vitro* experiments demonstrated that dapagliflozin inhibited the expression of *MCP-1* and *OPN* in mProx24 cells. These results indicate that dapagliflozin inhibits proinflammatory macrophage infiltration and inflammation in diabetic nephropathy.

Numerous studies have also reported an importance for oxidative stress and apoptosis in the pathophysiology of diabetic nephropathy [2], [26]. To investigate the effects of dapagliflozin in the etiology of diabetic nephropathy, Oxidative stress in the kidney was evaluated by assessing ROS generation. DHE staining revealed that compared with the non-diabetic *db/m* mice ROS were increased in the interstitia of the diabetic *db/db* mice. The intensity of DHE staining was lower in the dapagliflozin-treated *db/db* mice than in the control *db/db* mice. We also performed immunohistochemistry of Nox4 in diabetic kidney as a promoter of ROS generation. The fact that Nox4 expression was upregulated in diabetic *db/db* mice and decreased by the administration of dapagliflozin suggests that dapagliflozin may reduce oxidative stress by suppressing Nox4-derived ROS generation in the kidney of *db/db* mice. Furthermore, we evaluated apoptosis in the kidney by TUNEL staining and quantitative analysis of gene expression of proapoptotic factors. The number of diabetes-induced apoptotic cells was lower in the dapagliflozin-treated *db/db* mice compared with that in the control *db/db* mice. Similarly, the expression levels of *Caspase-12* and *Bax* were suppressed by the administration of dapagliflozin. Finally, we performed an *in vitro* experiment and revealed that dapagliflozin suppressed the high-glucose-induced ROS generation and *Nox4* expression in cultured mProx24 cells. Taken together, these findings suggest that dapagliflozin suppresses diabetes-induced oxidative stress and apoptosis in the kidney of *db/db* mice.

To date, no studies have evaluated the effect of SGLT2 inhibitors on the progression of diabetic nephropathy in detail, and only two studies have reported renoprotective effects of SGLT2 inhibitors. The first report has demonstrated that the SGLT2 inhibitor, tofogliflozin, reduced albuminuria and glomerular hypertrophy in *db/db* mice [22]. The second report has shown that luseogliflozin slowed the progression of diabetic nephropathy in a type 2 diabetic rat model [27]. However, neither inflammation nor oxidative stress in renal tissue or in cultured renal cells was examined in these studies. To the best of our knowledge, this is the first study to investigate the protective effects of an SGLT2 inhibitor on diabetic nephropathy by inhibiting inflammation and oxidative stress by both *in* *vivo* and *in* *vitro* experiments.

Vallon *et al.* have shown that SGLT2 knockout attenuated hyperglycemia and glomerular hyperfiltration, but not renal injury, oxidative stress and inflammation in the streptozotocin (STZ)-induced type 1 diabetes model [28]. There are two possibilities for the discrepancy between their results and ours. First, it is well known that STZ has toxicity and that STZ itself may affect the kidney and induce renal injury, oxidative stress and inflammation. Second, the glucose level was lower in the STZ-induced diabetic SGLT2-knockout mice than in the diabetic wild-type mice (300 vs. 470 mg/dl); however, it was still much higher than the normal level. The glucose level in their diabetic SGLT2-knockout mice was similar to that in our untreated *db/db* mice. Therefore, hyperglycemia per se may induce oxidative stress, inflammation and renal injury. A recent clinical study has reported that empagliflozin ameliorated hyperfiltration, but not the urine albumin/creatinine ratio in patients with type 1 diabetes [29]. The treatment period in this study was only 8 weeks, which is too short to expect the effect of an SGLT2 inhibitor to reduce albuminuria. Furthermore, we should be careful not to administer SGLT2 inhibitors to type 1 diabetic patients, as SGLT2 inhibitors are indicated in patients with type 2 diabetes mellitus at present.

Tahara *et al*. have reported that the SGLT2 inhibitor, ipragliflozin, reduced plasma and liver levels of oxidative stress biomarkers and inflammatory markers, and ameliorated hyperglycemia in a mouse model of diabetes [30]. Chen *et al*. have shown that the SGLT2 inhibitor, BI-38335, suppressed the gene expression of inflammatory cytokines in pancreas, and improved glycemic control in *db/db* mice [15]. However, the effects of SGLT2 inhibitors on oxidative stress and inflammation in diabetic nephropathy were not investigated in these studies. To elucidate the precise mechanisms by which dapagliflozin inhibits diabetes-induced inflammation and oxidative stress, and thus ameliorates diabetic nephropathy, further investigations are needed.

In conclusion, we demonstrated that the SGLT2 inhibitor, dapagliflozin, ameliorates the characteristic changes of diabetic nephropathy and reduces albuminuria, as well as hyperglycemia and β-cell damage in *db/db* mice. Dapagliflozin shows renoprotective effects through its glucose lowering effect and at least in part by anti-inflammatory/oxidative stress effects in the diabetic kidney. Our results indicate that dapagliflozin may be a therapeutic option for the management of diabetic nephropathy.
